# Distribution of Pyrethroid Resistant Populations of *Triatoma infestans* in the Southern Cone of South America

**DOI:** 10.1371/journal.pntd.0004561

**Published:** 2016-03-23

**Authors:** Marinely Bustamante Gomez, Liléia Gonçalves Diotaiuti, David E. Gorla

**Affiliations:** 1 Laboratório de Referência em Triatomíneos e Epidemiologia da Doença de Chagas, Centro de Pesquisas René Rachou—FIOCRUZ Minas, Belo Horizonte, Brazil; 2 Instituto Multidisciplinario de Biología Vegetal (IMBIV), CONICET-Universidad Nacional de Córdoba, Córdoba, Argentina; Universidad de Buenos Aires, ARGENTINA

## Abstract

**Background:**

A number of studies published during the last 15 years showed the occurrence of insecticide resistance in *Triatoma infestans* populations. The different toxicological profiles and mechanisms of resistance to insecticides is due to a genetic base and environmental factors, being the insecticide selective pressure the best studied among the last factors. The studies on insecticide resistance on *T*. *infestans* did not consider the effect of environmental factors that may influence the distribution of resistance to pyrethroid insecticides. To fill this knowledge gap, the present study aims at studying the association between the spatial distribution of pyrethroid resistant populations of *T*. *infestans* and environmental variables.

**Methodology/Principal Findings:**

A total of 24 articles reporting on studies that evaluated the susceptibility to pyrethroids of 222 field-collected *T*. *infestans* populations were compiled. The relationship between resistance occurrence (according to different criteria) with environmental variables was studied using a generalized linear model. The lethal dose that kills 50% of the evaluated population (LD_50_) showed a strong linear relationship with the corresponding resistance ratio (RR_50_). The statistical descriptive analysis of showed that the frequency distribution of the Log (LD_50_) is bimodal, suggesting the existence of two statistical groups. A significant model including 5 environmental variables shows the geographic distribution of high and low LD_50_ groups with a particular concentration of the highest LD_50_ populations over the region identified as the putative center of dispersion of *T*. *infestans*.

**Conclusions/Significance:**

The occurrence of these two groups concentrated over a particular region that coincides with the area where populations of the intermediate cytogenetic group were found might reflect the spatial heterogeneity of the genetic variability of *T*. *infestans*, that seems to be the cause of the insecticide resistance in the area, even on sylvatic populations of *T*. *infestans*, never before exposed to pyrethroid insecticides, representing natural and wild toxicological phenotypes. The strong linear relationship found between LD_50_ and RR_50_ suggest RR_50_ might not be the best indicator of insecticide resistance in triatomines.

## Author Summary

The elimination of *T*. *infestans* in wide areas of the Southern Cone countries of South America and good results in other vector control initiatives showed the high susceptibility of triatomines to pyrethroid insecticides. Despite the constant efforts of vector control, the success was not complete in several areas of the Gran Chaco region of Argentina, Bolivia and Paraguay and parts of the Inter-Andean Valleys of Bolivia, and southern Peru, where persistent populations of domestic and wild *T*. *infestans* still persist. Additionally, high levels of insecticide resistance leading to control failures were described in the biogeographic region of the Gran Chaco, within the area of persistent house reinfestation after insecticide application. The influence of environmental variables on the geographic distribution of triatomine was previously studied for several species, showing significant correlations between Triatominae species occurrence and a number of environmental variables. We investigated the association between the spatial distribution of pyrethroid resistant populations of *T*. *infestans* and environmental variables. This study shows that pyrethroid resistance in *T*. *infestans* causing control failures is a highly localized event, spatially associated with the putative dispersion origin of the species, the location of the intermediate cytogenetic group, and with a particular combination of environmental variables, near the border between Argentina and Bolivia. The strong linear relationship found between LD_50_ and RR_50_ suggest RR_50_ might not be the best indicator of insecticide resistance in triatomines.

## Introduction

Chagas disease is the most important vector-borne infection in Latin America, affecting approximately 5–6 million individuals [1]. The disease is caused by the protozoa *Trypanosoma cruzi* (Trypanosomatidae) and the most frequent transmission mechanism is through the feces of infected blood-sucking insects belonging to the subfamily Triatominae (Heteroptera: Reduviidae). The main vector of *T*. *cruzi* in the countries of the southern cone of South America is *Triatoma infestans* (Klug). This species lives mainly in warm and dry rural areas and in close association with human dwellings, including domiciles and peridomiciliary structures [2, 3]. During the last years, a number of wild foci of *T*. *infestans* have been described, mainly in the Inter-Andean Valleys of Bolivia, in the Gran Chaco of Argentina, Bolivia and Paraguay [4–8] and in a Metropolitan region from Chile [9].

By 1960, the maximum geographic distribution of *T*. *infestans* occupied an estimated area of 6.28 million km^2^ [10], including parts of Argentina, Bolivia, Brazil, Chile, Paraguay, Peru and Uruguay. This species was responsible for well over half of the 18 million people infected by *T*. *cruzi*, as estimated by WHO [11] for the 1980 decade. After the establishment of the Southern Cone Initiative (INCOSUR) in 1991, wich had the main goal of interrupting the *T*. *cruzi* transmission using chemical insecticides to eliminate *T*. *infestans* populations and through blood transfusion, the vectorial transmission of *T*. *cruzi* was interrupted in Uruguay (1997), Chile (1999) and Brazil (2006), according to the certification of the Pan-American Health Organization (PAHO) [12]. In Argentina (seven provinces) and Paraguay (eastern) the transmission of *T*. *cruzi* was interrupted in several areas where the disease had been historically endemic [13]. The Departments of La Paz and Potosí in Bolivia recently certified the interruption of the vectorial transmission of *T*. *cruzi* [14]. As a consequence of the vector control interventions in the region, there was a significant reduction of the distribution area of *T*. *infestans* to less than 1 million km^2^, leading to a strong reduction of the new infections by *T*. *infestans* [10, 14–18].

Despite the constant efforts of vector control the success was not complete, *T*. *infestans* persists as domestic populations in several areas of the Gran Chaco region from Argentina, Bolivia and Paraguay and parts of the Inter-Andean Valleys of Bolivia, and southern Peru [19–21]. Persistent bug populations that survived the insecticide application at local spatial scales, were related with sources of peridomestic populations, operational failures, reduced residual effect of insecticide or development of resistance to pyrethroid insecticides that decrease the vector control efficacy [22–26]. Resistance to insecticides is a microevolutionary process, over which the dynamics, the structure of the population and the gene flow between groups of individuals would determine the maximum geographical spread of each process of resistance evolution [27, 28]. In different geographic areas of Argentina and Bolivia, resistance of *T*. *infestans* to pyrethroids was detected by 2000, [29–38]. The occurrence of insecticide resistance was relatively unexpected [39, 40]for a long life cycled-insect duration (compared with other pest species with insecticide resistance records), relatively low frequency of insecticide applications, unsustained-over-time, and low insecticide efficacy in the peridomestic structures that would leave many residual populations (not necessarily resistant, but susceptible individuals that were not affected by the insecticide that degraded before contacting the insects). Studies showed different toxicological profiles and mechanisms of resistance [31, 35, 36, 41]. High levels of insecticide resistance (populations that need 1000 times the amount of active ingredient to kill the same fraction of a susceptible population) leading to control failures were described in the biogeographic region of the Gran Chaco, coinciding with the area of persistent house reinfestation even after insecticide application.

The accumulation of evidence over the last years suggests that the occurrence of insecticide resistance in *T*. *infestans* populations is associated with the high genetic variability detected in the historical dispersion site of the species towards the southern cone of South America [21, 42, 43] and the strong spatial structure of the populations derived by low population dispersal rates [24, 44–45].

Why is it that control failure associated with insecticide resistance has only been recorded in this particular area and not anywhere else over the historical geographic distribution of *T*. *infestans*? The cause of the appearance of pyrethroid resistance is still under discussion. The repeated application of pyrethroid insecticide does not seem to be the only cause of pyrethroid resistance appearance, as resistant populations occurred in areas that received less insecticide pressure than others where resistance did not occur, and because multiple independent resistance mechanisms were detected in several populations. The diversity of resistance mechanisms and the genetic variability around the putative dispersion center of the *T*. *infestans* encouraged the consideration of the influence of environmental variables as another potential cause of pyrethroid resistance occurrence [28].

As far as we know, there is no demonstration of a causal relation between the effect of environmental variables and insecticide resistance, partly because of the difficulty of identifying the individual contribution of the genetic background and the abiotic factors. However, a number of studies have shown direct or indirect effects of environmental variables over the appearance of insecticide resistance in several insect pest species. For example, according to Foster et al [46] the selection for resistance to insecticides in *Myzus persicae* is subject to counteracting selection by cold, wet and windy conditions; whereas [47] showed that adaptive responses and DNA regions that control their expression have been linked to evolutionary responses to pollution, global warming and other changes. Interestingly, a significantly higher diapause propensities in carriers of the resistance alleles (37.0–76.2%) than in susceptible homozygotes (6.7%) was shown [48]. Although no diapause was ever shown to exist in Triatominae, it was shown that the developmental delays in fifth instar nymphs of Triatominae could be due to an adaptive risk-spreading diapause strategy [49], if survival throughout the diapause period and the probability of random occurrence of ‘‘bad” environmental conditions are sufficiently high.

The influence of environmental variables on the geographic distribution of triatomine was studied for several species, showing significant correlations between a number of environmental variables (particularly temperature) and species occurrence e.g. [10, 50]. As other phenotypic characters, the different toxicological profiles and mechanisms of resistance to insecticides is due to a combination of a genetic base and environmental factors [28], with the selective insecticide pressure being the best studied among the last factors. So far, studies on insecticide resistance on *T*. *infestans* did not consider the effect of environmental factors, that may influence the distribution of resistance to pyrethroid insecticides in *T*. *infestans* populations.

Guided by the question about the particular occurrence of vector control failures caused by pyrethroid resistance in this particular area, we explored in this study for the first time the geographic distribution of pyrethroid resistance of *T*. *infestans* populations and its association with environmental variables.

## Methods

### Data collection

An exhaustive compilation of all available data on studies about susceptibility *of T*. *infestans* to pyrethroid insecticides was carried out. Repeated data were discarded.

A database containing information on the field-collected specimens and methods used in the susceptibility studies based on topical application of insecticide was created. The database includes collection location coordinates, collection ecotope (intradomestic/peridomestic/sylvatic), value of the lethal dose that kills 50% of the evaluated population (LD_50_), resistance ratio 50 (RR_50_) (calculated as LD_50_ of the evaluated population/ LD_50_ of the susceptible population) and diagnostic dose (DD) (defined as percent mortality produced by twice the minimum concentration of the insecticide that causes 99% of mortality in the susceptible laboratory strain). All tests were carried out using first instar nymphs between 3–5 days, topicated with a 0.2 uL droplet applied with a Hamilton microsyringe.

Identifying a *T*. *infestans* population as resistant to pyrethroids is not easy, because no objective definition of resistance for triatomines exists. At least three criteria have been proposed to operationally define triatomines' resistance. Pan American Health Organization [51] defined as resistant all populations with RR_50_ > 5 (PAHO criteria from now on). Zerba and Picollo [52] suggested that a population should be considered resistant when RR_50_ > 2 (Z&P criteria from now on). WHO [53] proposed the use the DD and considered a population as resistant if mortality is < 80%, and susceptible if mortality > 98% (WHO criteria from now on), although the latter criteria is used mainly to evaluate resistance in mosquitos.

### Analysis of resistance occurrence

Using the three criteria mentioned above, *T*. *infestans* populations studied for pyrethroid resistance were classified as susceptible or resistant according to 7 different estimates of resistance-occurrence categories as follows. The first three categories derived directly from the three criteria mentioned above (namely, PAHO, Z&P, WHO). A fourth category (RR1) recorded as resistant any *T*. *infestans* sample that was classified as resistant by any of the three criteria. A fifth category (RR2) recorded as resistant any *T*. *infestans* sample that was classified as resistant by at least two of the three criteria. A sixth category (RR3) recorded as resistant a *T*. *infestans* sample that was classified as resistant by the three criteria. A seventh category (LD_50_) (strictly not a resistance category) considered the value of the LD_50_ (i.e. the amount of the active ingredient that produced 50% of mortality within the sample under study). It is worth remarking that RR1, RR2 and RR3 are derived variables from PAHO, Z&P and WHO variables, not independent of each other, as they are defined as “both” or “either”of the other criteria.

### Environmental data

The analysis of the association between resistance occurrence and environmental variables was carried out using the WorldClim dataset (http://www.worldclim.org) [54], that characterizes climatic conditions over the Earth surface between 1950–2000 in a grid format, with a pixel resolution of 1km. Variables included 19 bioclimatic statistics derived from monthly total precipitation, and monthly mean, minimum and maximum temperature (Bio1 to Bio19 described in full at http://worldclim.org/bioclim). Altitude above sea level was added to the climatic variables.

### Modelling resistance occurrence

The distribution of *T*. *infestans* resistance to pyrethroid insecticides occurrence was carried out using the species distribution modelling approach [55], with the geographic coordinates of resistance occurrence recorded as “presences”. We explored two different approaches on the consideration of “absences”. On one approach, we considered the coordinates of *T*. *infestans* populations defined as susceptible, and on the other approach (usual within the context of species distribution modelling [56]), we considered a random selection of 1000 background (pseudo-absence) points taken over the Gran Chaco region and Inter-Andean valleys, the area where *T*. *infestans* populations still persists after the successful interventions of the Southern Cone Initiative [18].

For the study of the association between environmental variables and resistance occurrence we used a binary response variable, assigning 1 to cases recorded as a site with a *T*. *infestans* resistant population, according to each of the seven resistance criteria mentioned above, and 0 to the susceptible populations or the randomly selected background points. The case of the LD_50_ data was analyzed similarly as a binary variable based on a threshold value that divided the dataset in high LD_50_ (assigned the value 1) and low LD_50_ (assigned 0) (see the appropriate section in Results for additional details). The analysis was based on a generalized linear model (GLM) with a logit link. Colinearity between bioclimatic variables was estimated using the variance inflation factor (*vif*), of the R *car* package. Only variables with *vif<*10 were considered for the construction of the model. The evaluation of the model was estimated using the partial area under the receiver operation curve, calculated with the pAUC package of R. Cross-validation (through the *cv*.*glm* function) was used to measure the robustness of model estimation. Data analysis was carried out with R (version 3.2.0).

In order to qualitatively explore the association between population genetics characteristics of *T*. *infestans* and the LD_50_ measured on the populations compiled in this study we used the geographic coordinates of the populations categorized by cytogenetics groups (andean, non andean and intermediate), as reported by [21].

## Results

**Fig 1:**
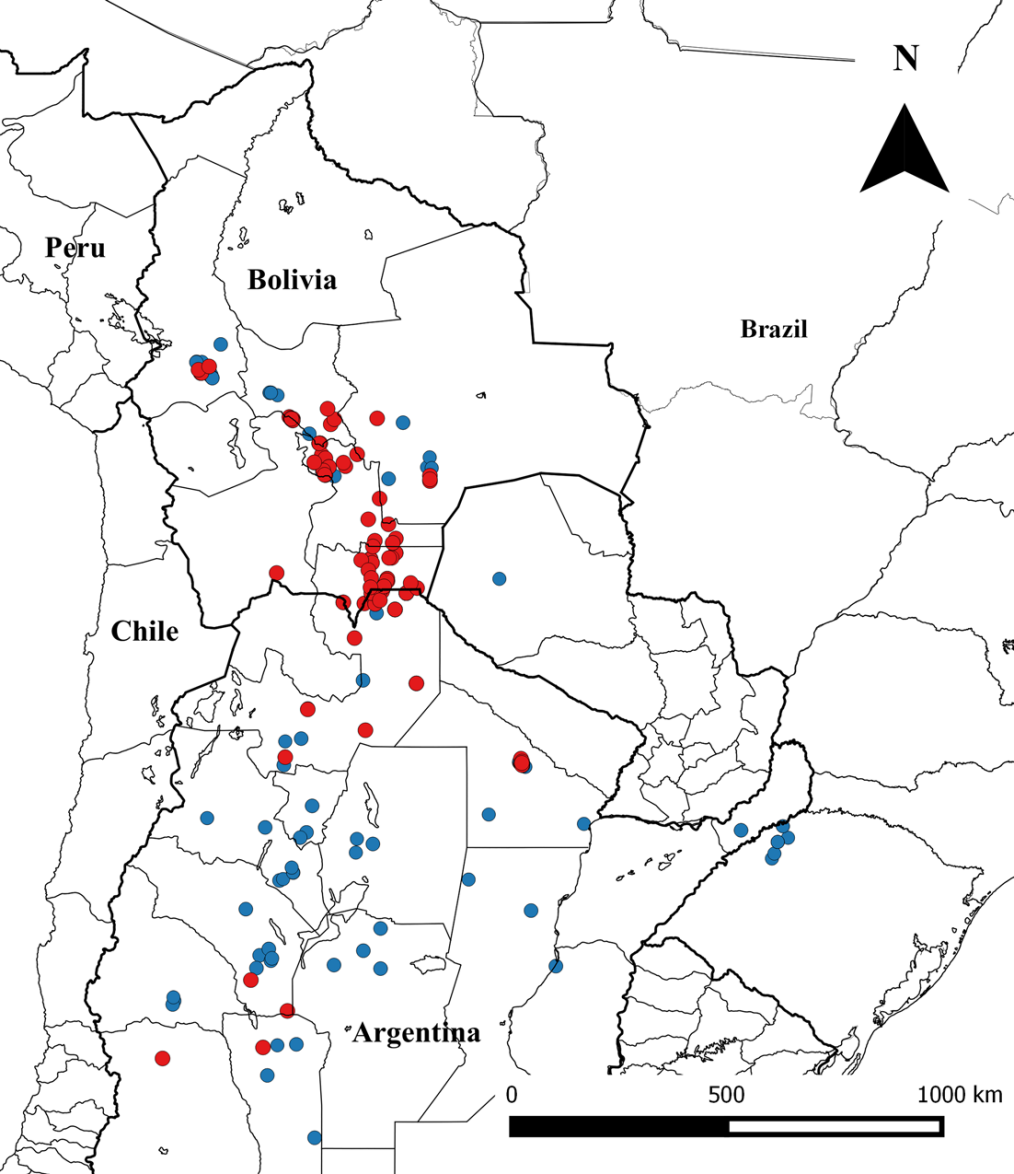
Geographic distribution of the 222 *T*. *infestans* populations evaluated for susceptibility to pyrethroids. Red circles: populations identified as resistant by PAHO and WHO criteria (RR_50_> 5 or mortality DD <80%); blue circles: populations identified as susceptible by PAHO and WHO criteria (RR_50_ < 5 or mortality DD > 80%).

The bibliographic compilation produced a total of 24 studies published since 2000, that evaluated 222 field-collected *T*. *infestans* populations for susceptibility to pyrethroid insecticides, from Argentina (101), Bolivia (106), Brasil (14) and Paraguay (1) (Fig 1). Not all studies reported RR_50_, or LD_50_ and DD, and as a consequence, the number of populations considered for the analysis was smaller than 222. Table 1 summarizes the occurrence of pyrethroid resistance, according to each criterium category. The highest recorded RR_50_ and LD_50_ were 1108 and 228, respectively from Campo Largo (Salta, Argentina). The complete dataset is included in S1 Table.

**Table 1:** Number of *T*. *infestans* populations studied for pyrethroid resistance according to each criterion (response variable) for resistance evaluation in triatomines.

Criteria (resp. variable)	Number of pyrethroid resistant populations	Number of pyrethroid susceptible populations	Total
PAHO	100	41	141
Z&P	119	22	141
WHO	68	83	151
RR1	162	55	217
RR2	24	117	141
RR3	6	74	80
LD_50_	48	93	141

Response variables represent different ways of identifying susceptible/resistant populations. PAHO: considers resistant populations with RR_50_ > 5. Z&P: considers resistant populations with RR_50_ > 2. WHO: considers resistant a population with mortality <80%. RR1: considers resistant a population that was classified as resistant by any of the three criteria mentioned above. RR2: considers resistant any population that was classified as resistant by two of the three criteria. RR3: considers resistant any population that was classified as resistant by the three criteria.

Reported values of LD_50_ show a significant and strong linear relationship with their RR_50_. However, detailed consideration of compiled data shows that all the reported values by Germano and colleagues [20, 32, 36, 23], together with one reported value by [30] and one by [23] (n = 33, all tests used deltamethrin, except [30], that used B-cyfluthrin) (*T*. *infestans* populations widely distributed around Argentina, Bolivia and Paraguay) lie over different line functions (with very low residual variability), compared with the rest of the n = 112 reported values by the other 15 authors (slope values of 7.5 and 4.4 for the first and second group, respectively; significantly different, P < 0.0001) (S1 Table and S1 Fig).

### Pyrethroid susceptibility and environmental variables

A first set of analysis for the first six categories using the recorded resistant and susceptible populations to fit the generalized linear model (GLM) with the environmental variables as predictors showed a low ability to explain the variability of the resistant populations (of any considered category) occurrence. Among the fitted models, the best one explained 43% of the resistance occurrence distribution, based on the PAHO criteria. This model was fitted using 100 points of resistance occurrence and 41 of susceptibility occurrence and included the highest number of environmental variables (Table 2). These 41 susceptibility points is a probably biased sample of the susceptible populations occurrence, driven by the special interest in the region of the border between Argentina and Bolivia; the actual distribution of susceptible populations is probably more widely distributed. A similar result was found when the observed susceptible populations were replaced by the set of 1000 randomly selected points taken from the entire Gran Chaco region and Inter-Andean valleys, where *T*. *infestans* populations are still patchily present. The analysis showed that none of the environmental variables (either considering the location of the susceptible population or taking background points) were able to account for more than 50% of the resistance occurrence, defined by each of the 6 mentioned criteria.

**Table 2:** Best GLM models for each response variable as a function of bioclimatic variables (Bioxx). Response variables are of binary type (0,1).

Response variable	Environmental variables (equation GLM BIOS)	Approx. R^2^	AUC	cv.glm
**1. PAHO**	-7.85 + 0.04 Bio2 + 0.01 Bio9–0.02 Bio13–0.25 Bio14 + 0.01 Bio18	0.43	0.83	0.08
**2. Z&P**	-2.52 + 0.08 Bio2–0.26 Bio3–0.001 Bio4 + 0.03 Bio9–0.02 Bio13 +0.02 Bio14 + 0.08 Bio15 + 0.009 Bio18	0.39	0.81	0.09
**3. WHO**	-2.63 + 009 Bio2–0.26 Bio3–0.001 Bio4 + 0.02 Bio9–0.01 Bio13–0.03 Bio15 + 0.01 Bio18	0.24	0.69	0.08
**4. RR1**	-7.6 + 0.04 Bio2–0.10 Bio3 + 0.02 Bio9–0.01 Bio13 + 0.05 Bio15 + 0.006 Bio18	0.27	0.73	0.13
**5. RR2**	-13.48 + 0.08 Bio2–0.13 Bio3 + 0.01 Bio8 + 0.02 Bio9 + 0.01 Bio19	0.18	0.50	0.04
**6. RR3**	-6.43 + 0.02 Bio9–0.004 Bio16	0.08	0.31	0.01
**7. Log (LD**_**50**_**)**	-25.93 +0.15Bio2–0.34 Bio3 + 0.07 Bio9 + 0.09 Bio15 + 0.006 Bio18	0.55	0.95	0.04

Approx. R^2^ = residual deviance/null deviances; AUC = Area Under the Receiver Operator Curve; cv.glm = Estimated error Cross-validation from generalized linear model.

Bio2 = Mean Diurnal Range (Mean of monthly (max temp—min temp)); Bio3 = Isothermality (Bio2/Bio7) (* 100); Bio4 = Temperature Seasonality; Bio8 = Mean Temperature of Wettest Quarter; Bio9 = Mean Temperature of Driest Quarter; Bio13 = Precipitation of Wettest Month; Bio14 = Precipitation of Driest Month; Bio14 = Precipitation of Driest Month; Bio15 = Precipitation Seasonality (Coefficient of Variation); Bio16 = Precipitation of Wettest Quarter; Bio18 = Precipitation of Warmest Quarter; Bio19 = Precipitation of Coldest Quarter.

### The geographic distribution of LD_50_

The descriptive analysis of LD_50_ values, showed that the frequency distribution of the Log (LD_50_) is bimodal, suggesting the existence of two statistical subpopulations (groups).

**Fig 2:**
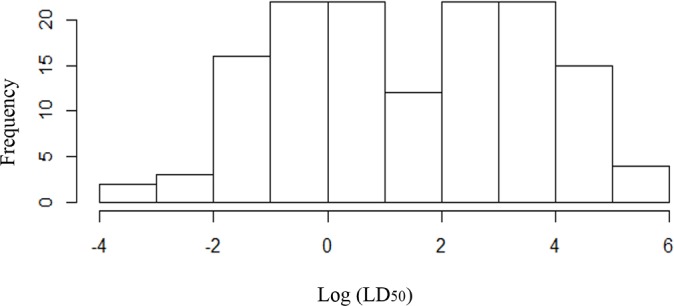
Frequency distribution of Log (LD_50_) in the *T*. *infestans* studied populations (n = 141). The value Log (LD_50_) = 2.6 (equivalent to 13.6 ng a.i./insect) is the threshold value that best separates the two subpopulations.

The value 2.6 is the threshold value that best separates the two groups. Calculating the descriptive statistics separately for the two groups, the group with lower Log (LD_50_) has an average = 0.17 and standard deviation = 1.47, whereas the group with higher Log (LD_50_) has values 3.82 and 0.74, respectively (Fig 2).

Driven by this identified pattern, we plotted the distribution of *T*. *infestans* populations classifying them in two groups, with low (≤ 2.6) and high (> 2.6) Log (LD_50_). The geographic distribution of these groups show a particular concentration of populations with highest LD_50_ over the region identified as the putative center of dispersion of *T*. *infestans*. Thus, we pursued the analysis classifying the two LD_50_ groups assigning 0 to those showing Log (LD_50_) ≤ 2.6 and 1 to those showing Log (LD_50_) > 2.6.

The analysis of the geographic distribution of these two *T*. *infestans* populations based on the Log (LD_50_), with 2.6 as the threshold value, showed a significant fit of the GLM model with the environmental variables as predictors. The model was based on 48 population samples where the Log (LD_50_) > 2.6 and 92 population samples where Log (DL_50_) was < 2.6. After variable selection to eliminate colinearity (*vif*<10), a model including 5 significant environmental variables was able to explain 55% of the variation in the distribution of the Log (LD_50_) groups (Table 2). The environmental variables Mean Diurnal Range (Mean of monthly (max temp—min temp)) (Bio2), Mean Temperature of the Driest Quarter (Bio9), Precipitation Seasonality (Coefficient of Variation) (Bio15); Precipitation of the Warmest Quarter (Bio18) are positively correlated, whereas isothermality (Bio2/Bio7) (* 100) (Bio3) is negatively correlated with the occurrence of high LD_50_ populations.

**Fig 3:**
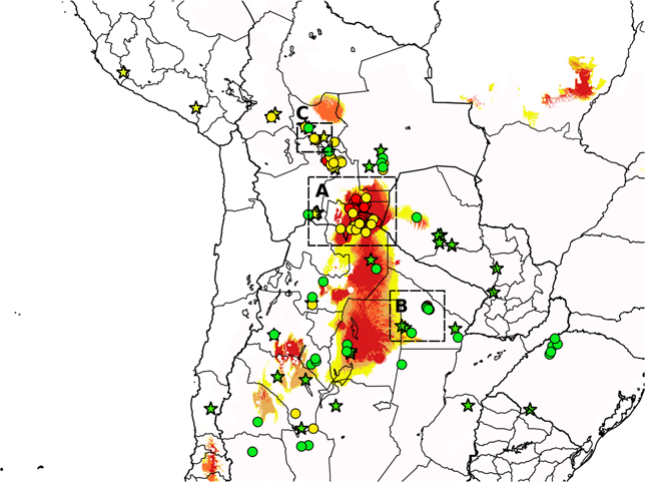
Location of *T*. *infestans* populations analyzed in the present study (circles) and those studied by [21] (stars). Green, yellow and red stars: Non-andean, Andean and Intermediate cyto-genotypes, respectively (sensu [21]. Green (susceptible populations), yellow and red (resistant populations) circles indicate ranges of Log (LD_50_): -4.0–-0.15, -0.15–2.6, 2.6–5.4, respectively. Coloured background indicates the gradient of log(DL_50_) predicted by the GLM (see text): yellow: Log(LD_50_) >2, to red: Log (LD_50_)> 2.6. A, B and C rectangular areas are enlarged in S2, S3 and S4 Figs.

Using the model describing the distribution of populations with low and high Log (LD_50_), a map with the potential distribution of populations with highest LD_50_ values was created (Fig 3). The area identified as the one where *T*. *infestans* populations could show highest LD_50_ includes the border between Bolivia and Argentina (see S2 Fig), and southward to the east of Salta and north central Santiago del Estero provinces (Argentina). The model predicts a disjunction area towards the border of La Rioja and San Juan provinces (Argentina) and towards the north of the Cochabamba Department (Bolivia) (see S3 Fig). The model fails at describing the occurrence of one highly resistant population (Log(LD_50_)>2.6) in Chuquisaca (-65.25, -19.05, a population studied by [28]) and 5 populations (out of 13) concentrated at the south of the Guemes Department (Chaco Province, Argentina, see S4 Fig). The location of the other 40 populations is correct. The model showed a high goodness of fit, with an, AUC = 0.95, pROC = 77.8 (61.9–90.9) and highly robust, with only 3.6% error estimated by the leave-one-out cross validation method.

After identifying the significant model that described the distribution of high and low Log (LD_50_) groups, separated by the 2.6 threshold, we calculated 3 additional models, using 2, 2.2 and 2.4 as threshold values to separate low and high Log (LD_50_) groups. All models were significant, included the same environmental variables and explained over 50% of the variation of the geographic distribution of the newly defined groups. From each model, a map showing the prediction of high Log (LD_50_) occurrence was produced. Fig 3 shows the geographic distribution of the highest values of Log (LD_50_) in the four models defined by different threshold values (Log (LD_50_)>2.0 to Log (LD_50_)>2.6 with a step of 0.2).

A map of the distribution of the three *T*. *infestans* cytogenetic groups (andean, non-andean and intermediate reported by [21]) and the distribution of Log (LD_50_) measured on *T*. *infestans* populations shows an almost perfect match between the highly resistant *T*. *infestans* populations (Log (LD_50_)> 2.6) and the intermediate cytogenetic group (Fig 3).

## Discussion

Pyrethroid insecticides were introduced into the pest control market by the end of 1970, and were rapidly identified as a major tool for the control of agricultural pests and vectors of human diseases [56]. At present, pyrethroid insecticides have a 25% share of the insecticide market, and are used in different formulations in the public health sector because of their efficacy, toxicity profile, persistence and low impact over the environment [57–59].

Pyrethroids were incorporated as a tool for the control of domestic triatomines by mid 1980s. The elimination of *T*. *infestans* in wide areas of the Southern Cone Countries of South America and good results in other vector control initiatives showed the high susceptibility of triatomines to pyrethroids [21, 39]. The reduction of house infestation by *T*. *infestans* is a success story over about 90% of its maximum geographic distribution area. This success is backed by a long history of vector control programs effort that started in the mid 1950s and made the strongest advances through the INCOSUR, coordinated by PAHO. The main tool for the elimination of house infestation by *T*. *infestans* was the application of residual insecticides (particularly pyrethroids). However, other socio-demographic factors, such as rural-urban migration, improvement of house quality in rural areas, community education and/or land use changes had contributed to this trend [60].

Although high impact was obtained in the elimination of intradomestic populations of *T*. *infestans* in most parts of the southern cone of South America, houses of several rural communities in the Gran Chaco are still infested by *T*. *infestans*. A number of reasons have been mentioned to explain the persistence of *T*. *infestans* populations in the area, including low insecticide efficacy when applied to peridomestic structures, unsustainability of vector control interventions, and insecticide resistance. Low pyrethroid efficacy is caused by rapid degradation, as has been shown by field measurements of the residual activity of the insecticide sprayed over wood and adobe [61] and by a number of field studies that repeatedly recorded the persistence of frequent residual populations shortly after the insecticide spraying [62]. In addition to the low efficacy of pyrethroid insecticides, the unsustainability of vector control interventions allows the recovery of the even small residual populations of *T*. *infestans* [58, 63]. Nevertheless, the majority of the sustained vector control failures in the area still occupied by this vector can be attributed to the factors mentioned (low efficacy of pyrethroid insecticides and unsustainability of vector control interventions).

Resistance in the identified hot spot is higher than in other places and it is apparently independent of the frequency of insecticide application in the area, that is not different to the frequency of insecticide application elsewhere. Therefore, we propose that the occurrence of pyrethroid resistant populations in the border between Argentina and Bolivia is not a primary result of the insecticide selection pressure, but a consequence of the existence of naturally tolerant populations of *T*. *infestans*, shown by the occurrence of resistant *T*. *infestans* population of sylvatic origin. The resistance remains high not because of an insecticide-based selection process, but as a natural selection process acting over a population having a naturally high frequency of resistant individuals. A similar interpretation was produced in the review of Mougabure-Cueto and Picollo [28]. The compilation of studies on pyrethroid resistance in *T*. *infestans* analyzed in this study, shows that the frequency distribution of the Log(LD_50_) for pyrethroids is bimodal, with two well spatially separated statistical groups. This is the first time this resistance feature is shown. The significance of these two groups is not clear. It might reflect the spatial heterogeneity of the high genetic variability of *T*. *infestans*, that seems to be one possible cause of the insecticide resistance in the area, even on sylvatic populations of *T*. *infestans*, never before exposed to the pyrethroids, representing natural and wild toxicological phenotypes. The spatial heterogeneity of the LD_50_ is associated with a combination of 3 temperature- and 2 rainfall-derived environmental variables, as shown by the significant fit of the generalized linear model developed in this study. This is the first time the spatial heterogeneity of resistance is shown significantly associated with environmental variables. Panzera et al. [21] speculated that the intermediate cytogenetic group might have appeared recently, as before 1998 house infestation was very low (ranging between 0.5 and 0.8%). These authors suggest that since 1998, and despite continued vector control activities, there has been a gradual increase of insects in houses, reaching house infestation levels of 50 to 80% in 2004. An alternative explanation is that this intermediate cytogenetic group was already in the area and was revealed only after continued vector control activities over a population with high frequency of resistant individuals selected the most resistant ones.

If *T*. *infestans* showed widespread resistant populations, why is it that control failures have only been reported in a limited area of the *T*. *infestans* distribution, even though pyrethroid insecticides for the control of the species are in use for more than 30 years now? The vector control failure within a limited area might suggest that the resistance in areas outside of the problematic area is not increasing, even though pyrethroid insecticides are in use, at the same frequency during the last 20 years, or even at higher frequency as it occurred during the last decade in some provinces in Argentina [64].

The occurrence of independent resistance mechanisms suggests that the process is widespread, but that it is not evolving rapidly, as expected by the demographic features of the species. Resistance to pyrethroids is widespread over the arid chaco and Andean valleys of Bolivia, although the high level of pyrethroid resistance (and other active ingredients, such as fipronil) occurs around the putative center of dispersion of the species, where the genetic variability is very high, and a particular combination of environmental variables exists.

We do not have enough information about the process that lead to the occurrence of the highly resistant *T*. *infestans* populations in the hotspot, to produce a meaningful mechanistic model able to analyze the relation between the occurrence of resistant populations of *T*. *infestans* and environmental variables. This is a limitation of the study, that can not demonstrate a causal relation between pyrethroid resistance and environmental variables, because the model we based our study on is a statistic one. To be able to demonstrate a causal relationship, we would need a mechanistic model integrating population dynamics, population genetics and environmental variables. Regrettably, we were not able to find publications compiling a geographic database on population genetics characteristics, equivalent to our compilation on pyrethroid resistance, to carry out an equivalent study on the relationship between environmental variables and population genetics.

The compiled published data shows that the highly resistant *T*. *infestans* populations are geographically limited (except one location in central Chaco province (Argentina) and one north of Potosi (Bolivia)) within an environmental variable space that does not occur towards the north of Bolivia, but does occur south, down to Santiago del Estero in central Argentina. As we can not claim a causal relation between insecticide resistance and environmental variables, we can not use the resulting model to predict the occurrence of highly resistant *T*. *infestans* populations. However, we can identify the area highlighted by the model as the one that possesses a similar combination of environmental variable values to the one where the highly resistant *T*. *infestans* populations occurs. If there is a causal relation between environment and pyrethroid resistance, then the area identified by the model should be carefully considered as an area of potential occurrence of highly resistant *T*. *infestans* populations. An important consideration should be given to the fact that if insecticide resistance existed in the area, without the need of selection by insecticide pressure, even if the use of pyrethroids is stopped, the frequency of resistant individuals will remain high.

The analysis of the relation between RR_50_ and LD_50_ revealed the existence of two groups of populations in the compiled database. It is difficult to discern the cause of this discrepancy, as it could appear as a consequence of the use of different susceptible populations, or that the studied populations really have a different relation between RR_50_ and LD_50_. Additional studies on this relation could determine wether these two population groups are artifacts or not. More importantly, if shown that there is only one linear relationship between RR_50_ and LD_50_, the use of RR_50_ for resistance detection should be revised, as it would mean that LD_50_ multiplied by a constant (the slope) would give the RR_50_ value.

## Supporting Information

S1 Table Compiled database of studies on *Triatoma infestans* susceptibility to synthetic pyrethroids. All studies used topical application of 0,2 μL of the active ingredient on age and weight standardized first instar nymphs produced by adults collected on domestic, peridomestic or sylvatic ecotopes. PAHO, Z&P and WHO columns contains the binary codes (0, 1) identifying susceptible (0) or resistant (1) populations, according to the corresponding three criteria currently used to identify insecticide resistance in Triatominae. LD_50_: lethal dose 50, RR_50_: resistance ratio 50, DD: mortality (%).
(XLS)

S1 Fig Relation between RR_50_ and LD_50_ of 145 *T*. *infestans* populations. Red circles: data reported by Germano et al [20, 32, 36], Carvajal et al [23] and Picollo et al [30], linear function is RR_50_ = -2.02 + 7.53 LD_50_, R^2^ = 0.99, n = 33; black circles: data reported by the rest of the authors reported in the compiled database (S1 Table), linear function is RR_50_ = -2.76 + 4.41 LD_50_, R^2^ = 0.98, n = 112.
(TIFF)

S2 Fig Fig 1 zoomed on rectangle A, over the area where most of highly resistant *T*. *infestans* populations (Log (LD_50_) > 2.6) (red circles) and the intermediate cytogenetic group (red stars) are found. (TIF)

S3 Fig Fig 1 zoomed on rectangle B, over the Potosi Department (Bolivia) where two highly resistant *T*. *infestans* populations (Log (LD_50_) > 2.6) (red circles) are correctly identified by the GLM based on environmental variables. (TIF)

S4 Fig Fig 1 zoomed on rectangle C, over the Chaco province (Argentina) where a concentrated group of 5 highly resistant *T*. *infestans* populations (Log (LD_50_) > 2.6) (red circles) were not identified by the GLM based on environmental variables. (TIF)
