# Supplementary figures and images for: Distribution of Pyrethroid Resistant Populations of Triatoma infestans in the Southern Cone of South America
Source: PLoS Negl Trop Dis. 2016 Mar 23;10(3):e0004561. doi: 10.1371/journal.pntd.0004561 (PMC4805280; doi:10.1371/journal.pntd.0004561)

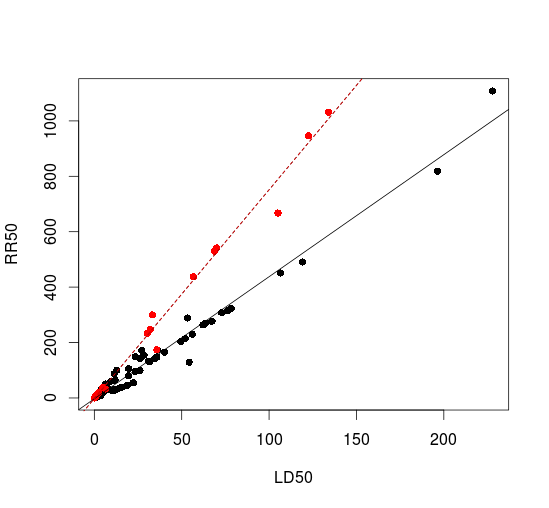

Supplement: S1 Fig — Red circles: data reported by Germano et al [20, 32, 36], Carvajal et al [23] and Picollo et al [30], linear function is RR50 = -2.02 + 7.53 LD50, R2 = 0.99, n = 33; black circles: data reported by the rest of the authors reported in the compiled database (S1 Table), linear function is RR50 = -2.76 + 4.41 LD50, R2 = 0.98, n = 112. (TIFF) [file pntd.0004561.s002.tiff]

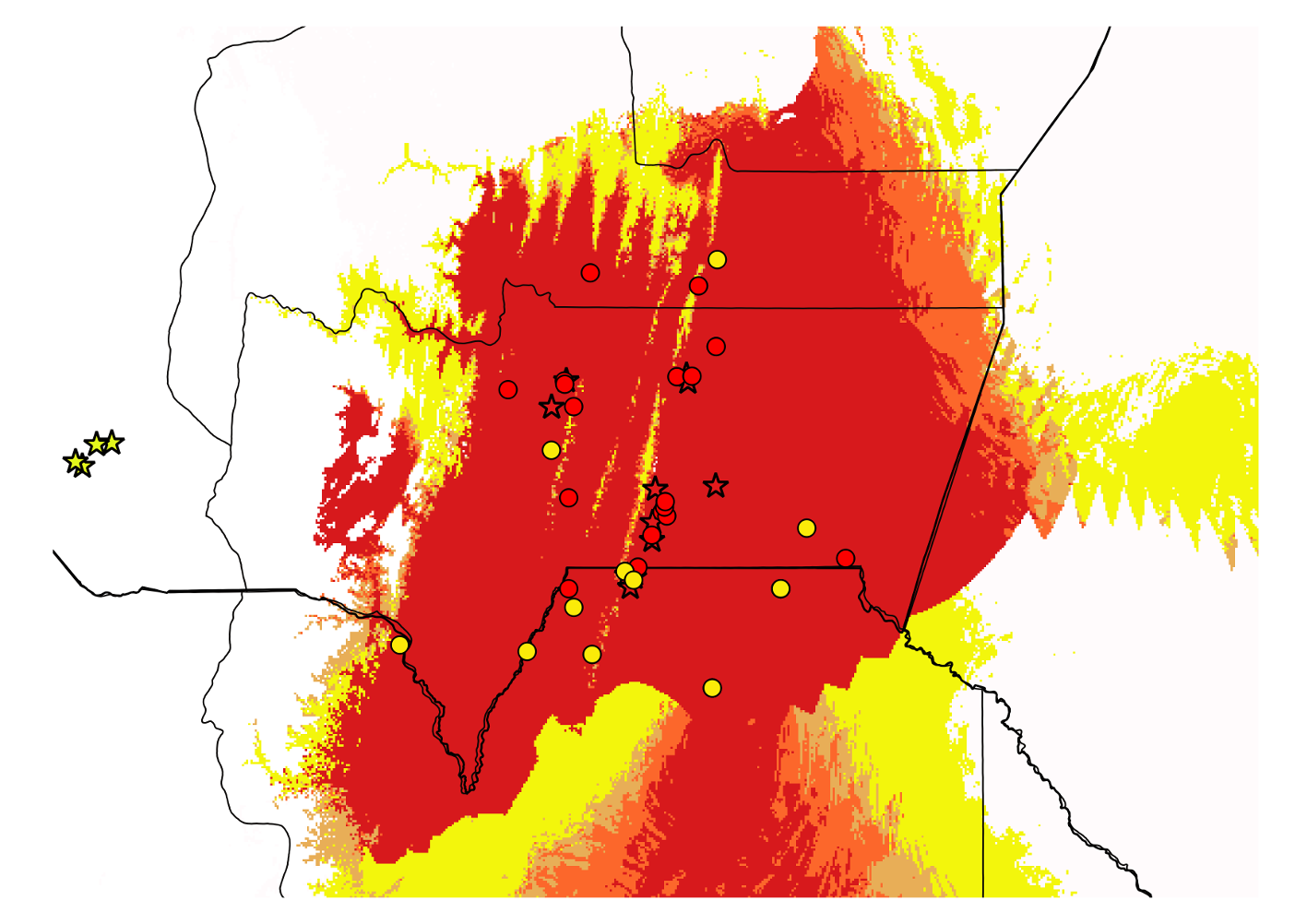

Supplement: S2 Fig — (TIF) [file pntd.0004561.s003.tif]

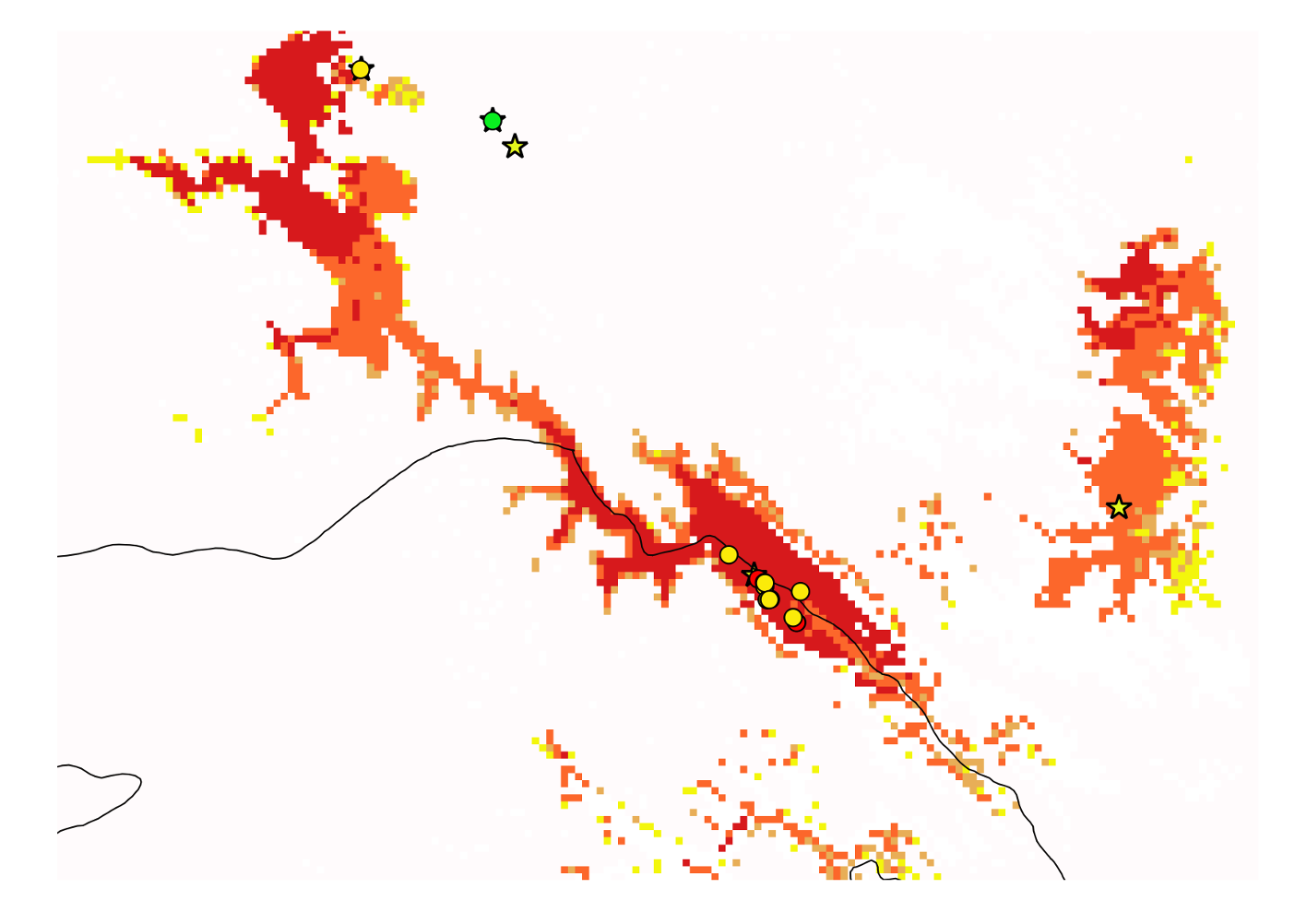

Supplement: S3 Fig — (TIF) [file pntd.0004561.s004.tif]

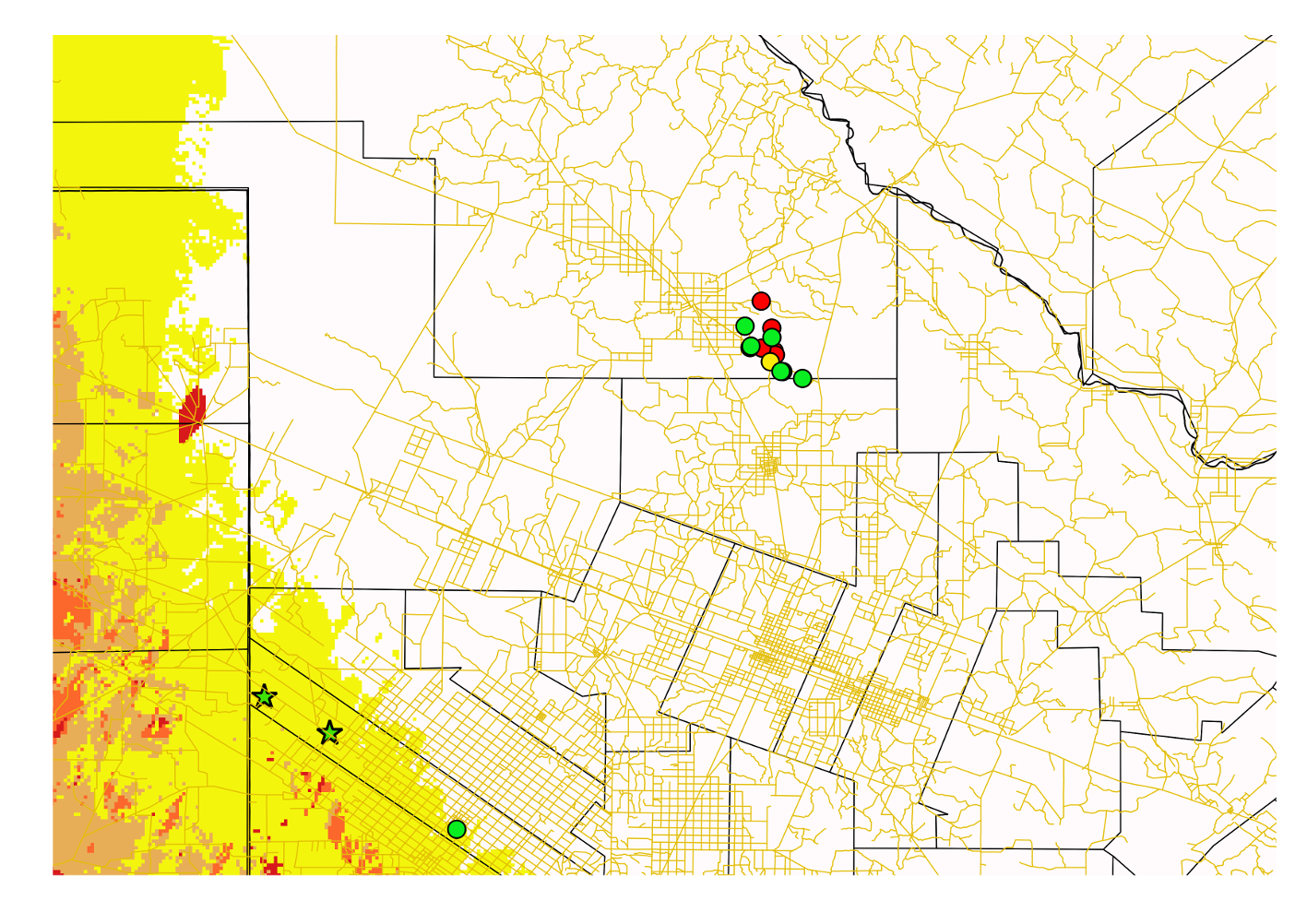

Supplement: S4 Fig — (TIF) [file pntd.0004561.s005.tif]
